# Validation of the Los Angeles Pre-Hospital Stroke Screen (LAPSS) in a Chinese Urban Emergency Medical Service Population

**DOI:** 10.1371/journal.pone.0070742

**Published:** 2013-08-07

**Authors:** Shengyun Chen, Haixin Sun, Yanni Lei, Ding Gao, Yan Wang, Yilong Wang, Yong Zhou, Anxin Wang, Wenzhi Wang, Xingquan Zhao

**Affiliations:** 1 Department of Neurology, Beijing Tiantan Hospital, Capital Medical University, Beijing, China; 2 Department of Neuroepidemiology, Beijing Neurosurgical Institute, Capital Medical University, Beijing, China; 3 Beijing Municipal Key Laboratory of Clinical Epidemiology, Beijing, China; 4 Beijing Emergency Medical Center, Beijing, China; Stanford University School of Medicine, United States of America

## Abstract

**Background and purpose:**

Early and accurate diagnosis of stroke by emergency medical service (EMS) paramedics is critical for reducing pre-hospital delays. The Los Angeles pre-hospital stroke screen (LAPSS) has been widely used as a validated screening tool for early identifying stroke patients by EMS paramedics. However, validation of LAPSS has never been performed in Chinese stroke population. This study is aimed to verify the LAPSS for early identifying stroke patients in a Chinese urban EMS.

**Methods:**

76 paramedics of five urban first aid stations attached to Beijing 120 EMS were involved. The paramedics were trained by professionals to quickly screen patients based on LAPSS. Potential “target stroke” individuals who met the base LAPSS screen criteria were identified. Sensitivity and specificity analyses of the LAPSS were calculated.

**Results:**

From June 10, 2009 to June 10, 2010, paramedics transported a total of 50,220 patients. 1550 patients who met the baseline screen criteria were identified as the potential “target stroke” population. 1130 patients had the completed LAPSS information datasheet and 997 patients were clinically diagnosed with stroke. The average time of completing the LAPSS was 4.3±3.0 minutes (median, 5 minutes). The sensitivity and specificity of the LAPSS in this study was 78.44% and 90.22%, respectively. After adjusting for age factor by excluding patients of >45 years old, the sensitivity was significantly increased to 82.95% with specificity unchanged.

**Conclusion:**

The paramedics of Beijing 120 EMS could efficiently use LAPSS as a screening tool for early identifying stroke patients. While the sensitivity of LAPSS in Chinese urban patient population was lower than those reported in previous LAPSS validation studies, the specificity was consistent with these studies. After excluded the item of “Age>45 years”, the sensitivity was improved.

## Introduction

Stroke has become one of the leading causes of death among all diseases in Chinese, which represents one fifth of the total population in the world. Increasing stroke incidence rate and the associated morbidity have resulted in a heavy social and economic burden to the Chinese healthcare system [1], [2], [3]. The advent of acute stroke therapies has highlighted the need for reliable emergency medical services (EMS) for stroke identification [4]. Through accurate diagnosis of stroke and subsequent transport with prior warning to appropriate stroke centers, EMS paramedics are in a critical position to reduce pre-hospital and in-hospital delays [5], [6]. Several pre-hospital stroke scales, including the Face Arm Speech Test (FAST), the Los Angeles Pre-hospital Stroke Screen (LAPSS), the Cincinnati Pre-hospital Stroke Scale (CPSS) and the Melbourne Ambulance Stroke Screen (MASS) have been established to quickly identify stroke for EMS paramedics. However, thus far none of these scales has been validated in Chinese population.

The LAPSS was designed, using a modified delphi approach, specifically for pre-hospital personnel. It consists of 4 history items, a blood glucose measure, and 3 examination items designed to detect unilateral motor weakness. Items were chosen not only to identify the most common acute stroke patients but also to exclude likely stroke mimics [7]. Despite the LAPSS has been validated in Western populations [8], [9], [10], the performance of the scale has not yet been examined in large Chinese stroke population. The aim of this study is to validate the LAPSS for identifying stroke individuals in patients accepted to a Chinese urban emergency medical service (EMS).

## Methods

**Figure 1:**
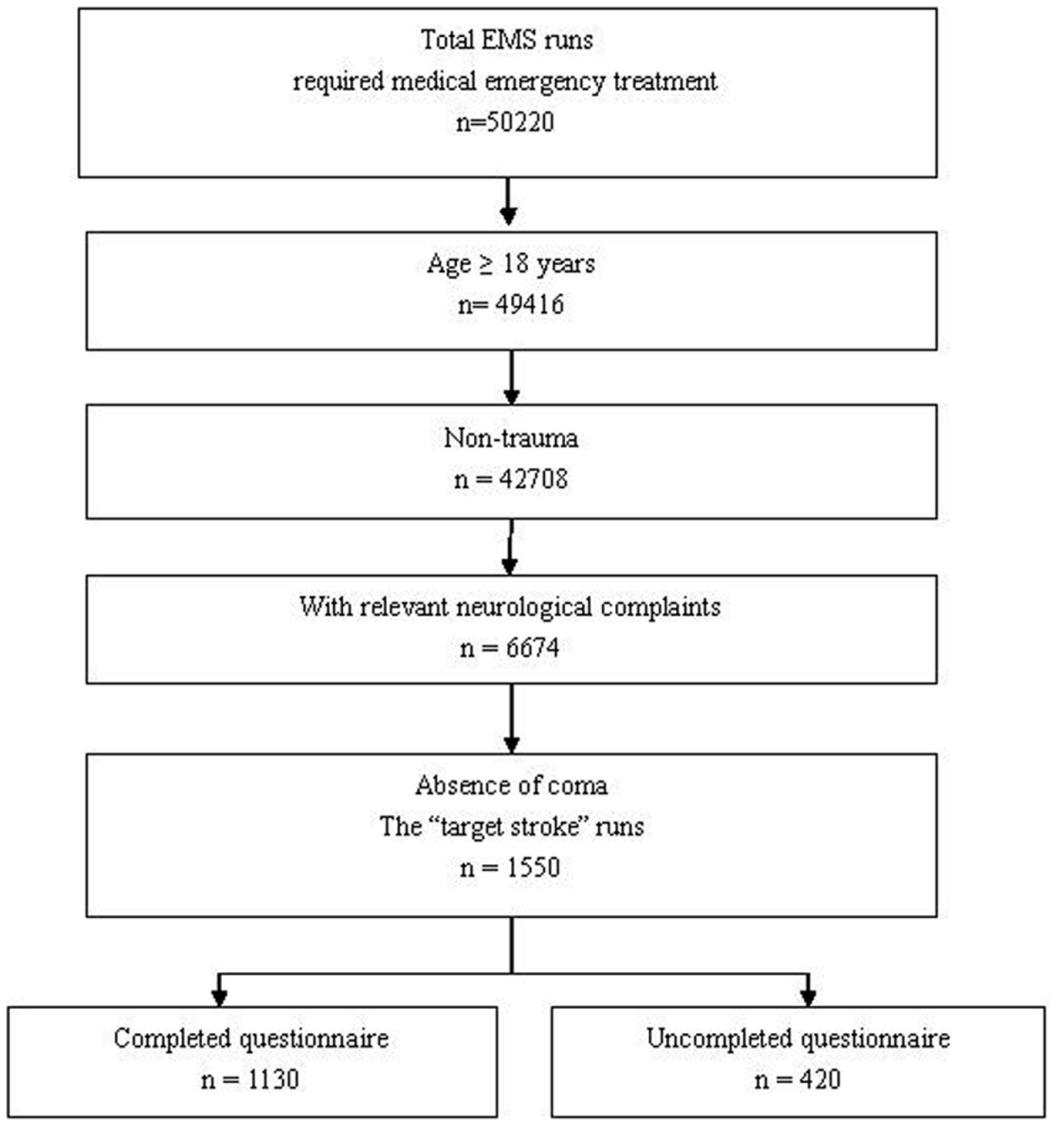
The flow chart of the method.

The Protocols conducted by the Department of Neurology, Beijing Tiantan Hospital and the Department of Neuroepidemiology, Beijing Neurosurgical Institute. The protocol was performed in the Beijing 120 Emergency medical service (EMS) systems. Methods are summarized in the Figure 1.

### Ethics Statement

Our study was approved by Ethics Committee at Beijng Tiantan Hospital affiliated to the Capital Medical University of China, in compliance with Declaration of Helsinki. All patients or their legal representatives signed informed consent form (ICFs).

### Emergency Medical System

The Beijing 120 EMS system is responsible for the emergency medical treatment and ambulance services in Beijing. It has a total of 65 first-aid stations located in different regions of Beijing. The study was performed at the five urban first-aid stations of 120 EMS, which are responsible for emergency transport service in Beijing central urban area, with 76 paramedics and 78 emergence vehicles involved. In a 180-minute LAPSS-based stroke training session, the 76 paramedics were trained by three experts of our study team (Shengyun Chen, Haixin Sun, Yan Wang) on how to accurately use the LAPSS for rapid identification of stroke during emergency transport of patients to 120 EMS stations. Qualification of the trained paramedics for grasping LAPSS skills was confirmed by post training tests.

### Survey Questionnaire

All questions of the survey scale were retrieved from the original LAPSS [7], [8] and translated into Chinese. It was presumed that translation does not influence validity or reliability of the scale since these questions are objective measurements.

### Subject

The paramedics were asked to identify the “target stroke” individuals from the patients who required the medical emergency treatment and were transported by the paramedic vehicles involved in the study during the enrollment period. The baseline screen criteria for the “target stroke” population were referred from the original LAPSS study including (1) age ≥18 years, (2) neurologically relevant complaints, (3) absence of coma, and (4) non-traumatic. The neurologically relevant complaints were identified with 6 categories, including (1) altered level of consciousness, (2) local neurological signs, (3) seizure, (4) syncope, (5) head pain, and (6) the cluster category of weak/dizzy/sick [8].

### Hospital Setting

Paramedics were instructed to complete a LAPSS assessment sheet in the “target stroke” runs. The “target stroke” patients were then transferred to the nearest hospitals that are qualified for treating stroke patients with thrombolysis.

### Data Collection

Completed study questionnaire datasheets were transferred to the dispatching centre for analysis. The overall efficacy study sheets were monitored in blinded manner by 3 paramedics for correcting any possible documentation errors before the analysis. All the datasheets were collected by the department of neurology, Beijing Tiantan Hospital. For the “target stroke” runs, 2 blinded neurologists reviewed the emergency department charts, recorded final emergency department discharge diagnoses, and verified absence or presence of potential stroke symptoms. The gold standard for diagnosis of stroke was used to identify the stroke patients. The medical documents and neuroimaging records were reviewed before the final diagnoses were verified.

### Statistical Analysis

Data of the LAPSS sheet were imported into a Microsoft Access database, and the statistical analyses were performed with SAS software version 9.2(SAS Institute, Cary, NC, USA). The sensitivity, specificity, positive predictive value, negative predictive value, and accuracy for LAPSS performance were calculated. These variables were calculated for LAPSS from the completed runs.

## Results

### Use of the LAPSS

Data were collected from 10 June 2009 to 10 June 2010. During this period, paramedics participated in the study transported 50,220 patients who required medical emergency treatment. Of these, 1550 met the baseline screen criteria for non-traumatic, non-comatose neurological runs. The paramedics completed questionnaires for 1130 “target stroke” patients. In these patients, 997(88.2%) were clinically diagnosed with stroke (60.7% ischemic, 24.6% hemorrhagic, and 2.7% transient ischemic attack). The age of these patients ranged from 20 to 101 years with mean age of 68.9±13.8 years (median, 72 years). 684 (60.5%) of them were male and 446(39.5%) were female. The average time for completing the LAPSS was 4.3±3.0 minutes (median, 5 minutes).

### Sensitivity and Specificity Analysis

Numbers of stroke patients identified by LAPSS and verified by clinical diagnosis are summarized in Table 1. According to the table, there were 13 false positive and 215 false negative cases by LAPSS. Table 2 details the diagnostic values of LAPSS and shows that the sensitivity of LAPSS for identifying real stroke patients was 78.44%, the specificity was 90.22% and the accuracy was 79.82% (77.48–82.16) (Table 2). The positive predictive value (PPV) and negative predictive value (NPV) were 98.36% and 35.82%, respectively.

**Table 1:** The diagnosis of LAPSS and the clinical diagnosis.

The diagnosis of LAPSS	The clinical diagnosis		Total (No.)
	Stroke (No.)	Non-stroke (No.)	
Stroke	782	13	795
Non-stroke	215	120	335
Total	997	133	1130

**Table 2:** The diagnostic values of the LAPSS.

Diagnostic values	LPASS	LAPSS (excluded the age item)
Sensitivity	78.44%(75.88–80.99)	82.95% (80.61–85.28)
Specificity	90.22% (85.18–95.27)	90.22% (85.18–95.27)
Accuracy,%	79.82% (77.48–82.16)	83.80% (81.66–85.95)
Youden’s index, YI	0.69 (0.63–0.74)	0.73 (0.68–0.79)
Odds product, OP	33.57	44.90
Positive predictive value, PPV	98.36% (97.48–99.25)	98.45% (97.62–99.29)
Positive likelihood ratio, PLR	8.02 (4.78–13.46)	8.49(5.06–14.23)
Negative predictive value, NPV	35.82% (30.69–40.96)	41.38% (35.71–47.05)
Negative likelihood ratio, NLR	0.24(0.21–0.27)	0.19(0.16–0.22)

Values in parentheses are 95% CIs.

Analysis of the individual items of LAPSS and the false negative stroke patients identified by LAPSS, summarized in Table 3, showed that in the total 215 false negative stroke patients, 134 (62.3%) had no facial paralysis or arm strength weakness and 45 (20.9%) were above 45 years old. Of interest, when the age factor was adjusted by excluding “Age>45 years” from analysis, the sensitivity and the accuracy of the LAPSS diagnosis of stroke was improved to 82.95% and 83.80% respectively, while the specificity remained unchanged. The NPV was improved to 41.38% after adjusting for age (Table 2).

**Table 3:** Analysis between the individual items of LAPSS and the false negative stroke patients.

		No. (%) the false negative stroke patients	
The No. of the false negative stroke patients		215	
The individual items for LAPSS			
Age>45 years.	No		45(20.9%)
History of seizures or epilepsy absent?	No		0
Symptom duration less than 24 hours?	No		17(7.9%)
At baseline, patient is not wheelchair bound or bedridden	No		19(8.9%)
Blood glucose between 60 and 400 mg/dl?	No		0
Facial paralysis or arm strength weakness	No		134(62.3%)

## Discussion

This study, to the best of our knowledge, is the first for validating the LAPSS as a pre-hospital screening scale for identifying stroke patients in a Chinese patient population with relatively large sample size (1130). We also reported that utilization of LAPSS in Chinese population is feasible by showing the trained paramedics could completed LAPSS for the accepted “target stroke” patients efficiently within 4.3±3.0 minutes (median, 5 minutes). The “D’s of Stroke Care” include 8 stages: detection, dispatch, delivery, door, data, decision,drug and disposition [11]. Reduction of time between onset of symptoms and initiation of therapy seems to benefit patient outcome [12]. An accurate stroke identification and prenotification of the receiving hospital could reduce symptom-to needle time in case of ischemic stroke [13]. On average, there were approximate 53.0% residential people in China calling EMS for first aids when stroke symptoms onset [14]. Considering the acutely aggressive nature of stroke disease, it is therefore critical to reduce the gap between pre-hospital transport by EMS and medical treatment at hospital, during which the well-trained paramedics can use an established and validated quick screening scale for early identification of stroke patients. In this circumstance, the LAPSS has been introduced for being used in the pre-hospital care by the 2005 American Heart Association Guidelines for Cardiopulmonary Resuscitation and Emergency Cardiovascular Care [15].

Validation of LAPSS has been previously performed by different EMS systems in independent studies with varied sensitivities and specificities (Table 4) [8], [9], [10]. When comparing to these previous studies, we found some differences in diagnostic values of LAPSS. The sensitivity (78.44%, 95% CI 75.88–80.99%) and specificity (90.22%, 95%CI 85.18–95.27%) of our study were both lower than which were achieved in the LAPSS validation study (91% and 97%) [8]. In contrast, other two groups studying LAPSS have reported values of sensitivity (74 and 78%) and specificity (83 and 85%) for LAPSS [9], [10]. Compared to these 2 studies, our results demonstrated some similarities as well as differences. Specifically, despite the sensitivities were quite close, the specificity in our study was higher than these two previous validation studies where the sample sizes were smaller than our study.

**Table 4:** The Comparison about validation of the LAPSS in different studies.

Studies	N	Sensitivity	Specificity	Accuracy	PPV	Positive LR	NPV	Negative LR
Kidwel et al ^8^	206	91%(76–98)	97% (93–99)	96%(92–98)	86%(70–95)	31 (16–147)	98% 95–99)	0.09 (0–0.21)
Bray et al ^9^	100	78% 67–87)	85% (65–95)	80%	93% (83–98)	5.27 2.16–13.13)	59% 42–74)	0.26 (0,16–0.4)
Bergs et al ^10^	31	74% 54–94)	83% (62–100)	77% (63–92)	88% (71–100)	4.42 (1.21–16.12)	67% (43–91)	0.32 (0.14–0.70)
Our study	1130	78.44%(75.88–80.99)	90.22%(85.18–95.27)	79.82%(77.48–82.16)	98.36%(97.48–99.25)	8.02(4.78–13.46)	35.82%(30.69–40.96)	0.24(0.21–0.27)

Values in parentheses are 95% CIs. PPV = Positive predictive value; NPV = negative predictive value; LR = likelihood ratio.

There were 215 false negative stroke patients based on LAPSS criterion in our study. Analysis of the individual items of LAPSS and the false negative stroke patients revealed that 134 (62.3%) patients had no unilateral facial paralysis or arm strength weakness and 45(20.9%) patients were under 45 years of age. In addition to this, 17(7.9%) patients had symptom duration of more than 24 hours and 19(8.9%) patients were wheelchair bound or bedridden at baseline. The LAPSS was originally designed to allow pre-hospital personnel to rapidly identify the most frequent types of strokes while excluding common stroke mimics (e.g. seizure or hypoglycemia) or patients unlikely to qualify for, or benefit from, acute stroke interventions (e.g. those with symptom duration for more than 24 hours or wheelchair bound or bedridden at baseline) [7], [8]. It should be noted that that 134(62.3%) patients had no facial paralysis or arm strength weakness. In the baseline, patients with neurologically relevant complaints were enrolled in our study. Some neurological symptoms such as verbal disorder or lower limb may be the only manifestations when acute stroke onsets. As a result, examining only the items in LAPSS may lead to increase in missed identification and diagnosis of strokes, causing increased false negative rate. In other pre-hospital stroke screen scales, such as MASS and CPSS, testing of speech impairment is also included based on a normal conversation between medical professionals and patients [9], [16]. Some neurological symptoms, such as speech impairment, lower limb weakness and visual impairment should also be tested in the future studies.

We also noted that there were 45(20.9%) false negative stroke patients in this study who were excluded by the “Age>45 years” item of LAPSS. In the verified study, the baseline screen criteria of the “target stroke” population included the condition “age ≥18 years” and the age range of the enrolled patients was 20 to 101 years. Therefore, the “Age>45 years” item of LAPSS was an confounding factor which may decrease the accuracy of the screen scale. In view of this, we validated the scale after excluding the age item. The results show that the sensitivity was increased (78.44% to 82.95%) but the specificity did not change. The Negative likelihood ratio was lower. Although mortality rates in young adults with ischemic stroke are low compared with similar older patients, they still sustain clearly more deaths than the young in the general population [17]. Thus, accurate identification of young adults with stroke is also vital to improving the poor prognosis for stroke. Consequently, exclusion of the age item “Age>45 years” of LAPSS might be more appropriate for use in Chinese population, based on the data we presented in this study. Further studies are needed to validate this modified LAPSS in other Chinese cities and emergency medical services.

The results of this study should be interpreted carefully with consideration of some limitations associated with the study. First, in our study, the frequency of non-traumatic, non-comatose neurologic runs in all transports (1550 out of 50,220, ∼3.1%) is markedly lower than in the US LAPSS validation study (34% of all transports) [8]. One of the possible reasons might be that in Chinese EMS system, there is currently no standard pre-screen process to select the target stroke population during the phone call when patients seek for emergency medical assistance. Additionally, EMS has to respond to the requests by sending out ambulance regardless of neurological and non-neurological runs. Therefore, despite the large number of all transports (50220), the number of target stroke population eligible for LAPSS test is low (1550), which is different from that in US. Second, the study was conducted in five of urban first-aid stations of Beijing 120 EMS. Although it was a citywide study, it did not cover the rural regions of the great Beijing metropolitan area. Second, a 180-minute LAPSS-based stroke training session may not be enough. A long-term training session may be needed to improve the accuracy of the LAPSS. In support of this, a MASS study showed that an excellent initial improvement in the diagnosis of stroke by paramedics was achieved and sustained after 3 years of citywide education and MASS implementation [18]. The accuracy of the LAPSS may be improved after long time use. The persistent and expansive training of medical personnel for the pre-hospital stroke screen scales are urgently required in Chinese emergency service system. Moreover, other pre-hospital screen scales, such as MASS and CPSS should also be validated in Chinese emergency service system. Further studies are needed to determine the most adequate stroke scale or stroke identification system for Chinese paramedics and population.
